# Molecular Modification Strategies for Enhancing CO_2_ Electroreduction

**DOI:** 10.3390/molecules30143038

**Published:** 2025-07-20

**Authors:** Yali Wang, Leibing Chen, Guoying Li, Jing Mei, Feng Zhang, Jiaxing Lu, Huan Wang

**Affiliations:** 1 Shanghai Key Laboratory of Green Chemistry and Chemical Processes, State Key Laboratory of Petroleum Molecular and Process Engineering, School of Chemistry and Molecular Engineering, East China Normal University, Shanghai 200062, China; wyl198701@163.com (Y.W.); 51264300098@stu.ecnu.edu.cn (L.C.); 51274300097@stu.ecnu.edu.cn (G.L.); 52284300050@stu.ecnu.edu.cn (J.M.); 51264300109@stu.ecnu.edu.cn (F.Z.); jxlu@chem.ecnu.edu.cn (J.L.); 2 College of Chemistry and Chemical Engineering, Ningxia Normal University, Guyuan 756099, China; 3 Institute of Eco-Chongming, 20 Cuiniao Road, Chenjia Town, Chongming District, Shanghai 202162, China

**Keywords:** electrocatalytic CO_2_ reduction, molecular modification, modification strategies, effects of action

## Abstract

Electrocatalytic CO_2_ reduction reaction (CO_2_RR) is a crucial technology for achieving carbon cycling and renewable energy conversion, yet it faces challenges such as complex reaction pathways, competition for intermediate adsorption, and low product selectivity. In recent years, molecular modification has emerged as a promising strategy. By adjusting the surface properties of catalysts, molecular modification alters the electronic structure, steric hindrance, promotes the adsorption of reactants, stabilizes intermediates, modifies the hydrophilic–hydrophobic environment, and regulates pH, thereby significantly enhancing the conversion efficiency and selectivity of CO_2_RR. This paper systematically reviews the modification strategies and mechanisms of molecularly modified materials in CO_2_RR. By summarizing and analyzing the existing literature, this review provides new perspectives and insights for future research on molecularly modified materials in electrocatalytic CO_2_ reduction.

## 1. Introduction

With the intensification of global climate change and the energy crisis, the development of effective carbon dioxide (CO_2_) reduction technologies has become one of the most pressing challenges faced by contemporary society [1]. Among various CO_2_ conversion methods, electrocatalytic CO_2_ reduction (CO_2_RR) has gained significant attention due to its mild reaction conditions, high energy efficiency, ease of control, and potential for being driven by renewable energy sources [2]. CO_2_RR can convert CO_2_ into high-value chemicals, such as CO (an important basic chemical raw material), HCOOH (an important organic acid and hydrogen storage carrier), CH_4_ (an essential fuel and chemical feedstock), CH_3_OH (an emerging fuel), C_2_H_4_ (a basic petrochemical raw material), and C_2_H_5_OH (an important fuel, solvent, and chemical), making it a key technology for achieving carbon neutrality [3,4,5,6]. However, CO_2_ molecules possess high chemical stability, and their electrocatalytic reduction typically requires high overpotentials [7]. Additionally, the reaction often involves multiple proton-coupled electron transfer steps, and even C-C coupling, which complicates the reaction pathway. Moreover, the thermodynamic redox potentials of various CO_2_RR products are not significantly different from each other and are close to that of the hydrogen evolution reaction (HER). These make it particularly challenging to improve the conversion efficiency of CO_2_ and further enhance the selectivity for a single product [8,9,10]. To address these issues, in CO_2_RR, it is essential to develop advanced catalysts to steer the reaction pathway and thereby achieve high selectivity of a single product.

With the development of CO_2_RR technology, traditional catalysts (such as metals/alloys, metal oxides, metal–organic frameworks, and covalent organic frameworks) have achieved significant progress in CO_2_RR. However, these catalysts still suffer from certain limitations, including insufficient active sites, poor stability, and particularly low selectivity for a single product [11]. Currently, molecular modification strategies have emerged as a focal point in CO_2_RR research, as they can effectively regulate the surface properties of catalyst materials. By introducing functional molecules onto the catalyst surface, adjusting the electronic structure of active sites, modulating the local reaction environment, and enhancing the adsorption of specific intermediates through hydrogen-bond-like interactions, the reaction can be guided toward the desired direction [12,13]. In contrast, molecular modification can significantly enhance the activation of carbon dioxide and product selectivity, making it a highly promising strategy for improving catalytic efficiency.

In recent years, as research deepens, molecular modification has become an important area in CO_2_RR research due to their structural flexibility and tunable functionality. The central objective is to address the key challenges in CO_2_RR by enhancing the selectivity for a specific product, suppressing competitive HER. Molecularly modified strategies (such as functionalizing catalyst surfaces with thiols, amino acids, pyridine salts, imidazole compounds, or polymers) aim to optimize key reaction steps by introducing specific electronic effects (regulating the electron density of metal centers) and spatial effects (microenvironmental regulation), thereby improving the target product yield of CO_2_RR reactions [14,15]. Particularly, the combination of metals/metal compounds and organic molecules is considered an effective way to achieve the above-mentioned regulation, thereby promoting the selective production of target products [16,17]. Moreover, the development of molecular modification polymer-based materials has played an important role in enhancing the stability and reaction efficiency of catalysts. The advantages and importance of molecular modification in CO_2_RR are evident in several aspects. The potential advantages of molecularly modified CO_2_RR lie in the precision of its design. Firstly, molecular modification enables the precise design of the surface properties of the catalyst, allowing for more effective adsorption and activation of CO_2_ molecules, thereby improving the reaction rate. Secondly, molecular modification aids in the selective regulation of the adsorption and conversion of intermediates in the CO_2_ reduction process, reducing side reactions and increasing the selectivity and yield of the desired products. Additionally, molecularly modified materials offer excellent tunability and diversity. Researchers can further optimize catalyst performance by altering the structure of organic molecules, the types of functional groups, and the arrangement of ligands, to adapt to different reaction conditions and target product requirements. Current molecular modification strategies mainly include metal–organic molecule bonding modification, weak interaction modification, and polymer-based modification. As an innovative catalyst optimization strategy, molecular modification has gradually become an indispensable part of CO_2_RR research. This paper will systematically review the main strategies of molecular modification and the mechanisms of molecularly modified materials in electrocatalytic CO_2_ reduction and explore how the design of molecularly modified materials can be further improved to enhance the performance of CO_2_RR, thus advancing the breakthroughs in CO_2_ resource utilization technologies.

## 2. Major Strategies of Molecular Modification

Molecular modification can regulate the electronic structure and geometric configuration of the catalyst surface and its interaction with the reactants, which in turn affects the adsorption, activation, and subsequent conversion pathways of CO_2_. To date, a variety of molecularly modified catalysts have been developed for CO_2_RR, including metal-based materials modified with organic small molecules, metal–organic frameworks (MOFs) and covalent organic frameworks (COFs) with functionalized ligands, and polymer modification materials, etc. Their strategies have been summarized for the molecular modification, such as metal–organic bonding, weak interactions, and modification with polymer-based materials.

### 2.1. Metal–Organic Molecular Bonding Modification

The most common approach for metal–organic molecular bonding is the connection of organic molecules to the surface of metal catalysts via metal-sulfur/nitrogen/carbon (M-S/N/C) bonds. Typical organic molecules employed for this strategy include thiols, amines, pyridines, etc. These molecules form stable bonds with metals, thereby modulating the metal′s electron density, surface morphology, and catalytic activity.

**Figure 1:**
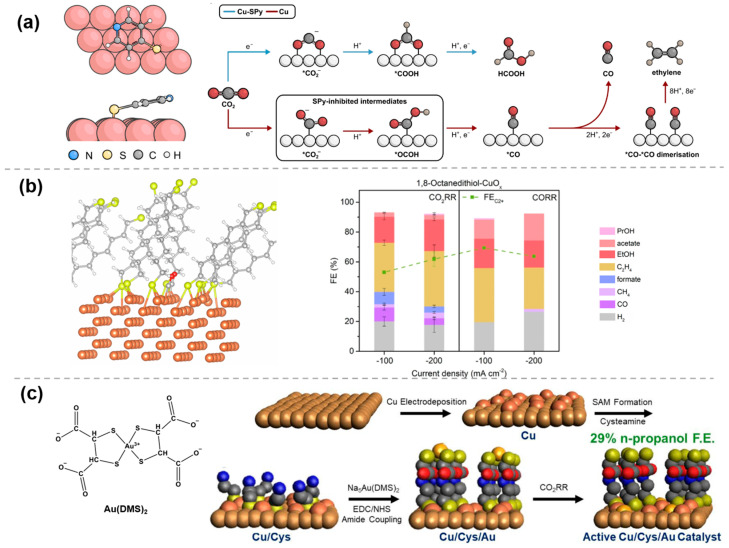
Modification of metal and organic molecules by M−S bonding: (**a**) Cu−SPy [18], with permission from John Wiley and Sons, copyright 2022; (**b**) 1,8−octanedithiol−CuO_x_ [23], with permission from Springer Nature, copyright 2025; and (**c**) Cu/Cys/Au catalyst [26], with permission from John Wiley and Sons, copyright 2025.

The most commonly used organic molecules are thiols, which primarily anchor to the catalyst surface through covalent bonding or chemical adsorption of the thiol groups. Once the-SH groups form bonds with the metal, the electrocatalytic CO_2_RR performance of the catalyst is effectively tuned. For example, through self-assembly in organic solution, 4-mercaptopyridine (SPy) molecules can be immobilized on the surface of Cu nanoparticles, to provide rational control over the product distribution. Fourier transform infrared (FTIR) spectroscopy and X-ray photoelectron spectroscopy (XPS) verified the successful immobilization, and density functional theory (DFT) calculations showed that the S atom preferentially binds to the two Cu atoms, with the ring slightly deviating from the Cu surface (Figure 1a) [18]. The presence of SPy energetically hinders the formation of the intermediate *OCOH, thereby blocking the CO production pathway, while the intermediate *COOH in the formic acid generation pathway remains unaffected, promoting formic acid production. Similarly, by using the interaction between sulfur and metals, thiols or sulfur-containing molecules can be modified onto the surface of other metal substrates, such as Au [19] and Ag [20], or used to stabilize nanoclusters [21,22]. The modified organic molecules on metals can modulate the supply of protons, the binding energy of key intermediates, etc., and thus enhance the selectivity of the targe product.

In addition to metals, thiol molecules can also be modified onto the surface of metal oxides to enhance CO_2_RR performance by modulating the metal–organic interface. Recently, Wang et al. [23] systematically examined and analyzed the structure–activity relationship with 180 molecular modifiers using an automated electrocatalysis platform and machine learning techniques. It was found that organic modifiers, such as 1,8-octanedithiol, formed metal–organic interphases over 10 nm thick on the surface of the CuO_x_ catalysts, instead of the traditional monolayer adsorption. These thick interphases significantly altered the interfacial structure of the catalyst, affecting the adsorption and conversion of CO_2_RR intermediates, thereby enhancing the selectivity of ethanol and C_2_+ products (Figure 1b). This strategy is also applicable to the development of electrocatalytic CO reduction (CORR) catalysts. Both SPy-modified Cu_2_O nanocubes [24] and thiol-modified Cu catalysts [25] display high FE in CORR to acetate.

Further, through the fine surface engineering modulation, the second metal nanoparticles can be loaded on the surface of the self-assembled monolayers, which facilitates highly selective C-C coupling via the synergistic effect of the bimetals as well as the uniformly distributed bimetallic active sites. For example, Barilex et al. [26] proposed a Cu/Cys/Au catalyst structure that significantly increased FE for n-propanol production. To fabricate this catalyst, a rough Cu layer was first electrodeposited on polycrystalline copper foil to enhance surface area. Subsequently, a cysteamine (Cys) self-assembled monolayer (SAM) was anchored to the electrodeposited Cu layer via Cu-thiolate bonds. Then, a Au^3+^ complex of dimercaptosuccinate (DMS) was attached to the Cys SAM through an amide coupling reaction. During the initial stage of CO_2_RR, Au^3+^ was reduced to Au and electrodeposited on the Cu surface, forming the final active Cu/Cys/Au catalyst (Figure 1c). At −1.2 V vs. reversible hydrogen electrode (RHE), the FE for n-propanol reached 29.1%, showing a significant improvement compared to traditional Au-Cu electrocatalysts.

**Figure 2:**
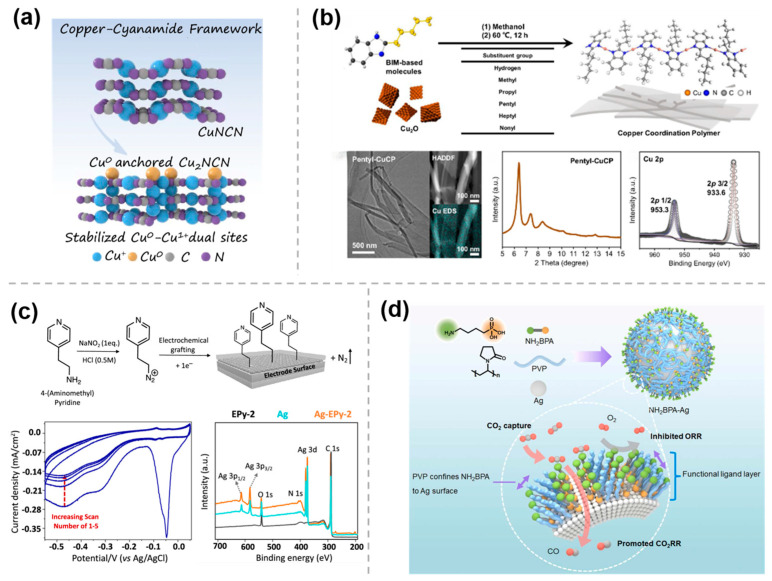
Modification of metal and organic molecules by M−N/C/O bonding: (**a**) Cu^δ+^NCN [27], with permission from Springer Nature, copyright 2024; (**b**) pentyl−CuCP [28], with permission from Springer Nature, copyright 2024; (**c**) Ag−EPy [29], with permission from American Chemical Society, copyright 2022; and (**d**) NH_2_BPA−Ag [30], with permission from Elsevier, copyright 2024.

The M-N bond has been extensively employed in organic chemistry to synthesize a wide range of organometallic complexes with rich structural diversity and versatile properties. Moreover, it can also serve as a fundamental structural motif for the preparation of organic molecule-modified materials, which has been shown to significantly enhance the catalytic performance of the resulting materials. To improve the selectivity and yield of multi-carbon products, Yan et al. [27] prepared a cyanamide coordinated isolated Cu framework (Cu^δ+^NCN), using a modified liquid-phase precipitation method. In this catalyst, the linear [NCN]^2−^ anions in the Cu^δ+^NCN open framework stabilize the Cu^0^-Cu^1+^ ensembles by strong covalent interactions and the fast electrons transfer, while Cu^δ+^ is linked to the nitrogen atom of [NCN]^2−^ and oxygen atom through Cu-N/C/O bonding to form a stable localized charge environment (Figure 2a). This distinctive coordination mode is key to achieving high ethylene selectivity (FE > 75%) and long-term stability (80 h). Furthermore, Wang et al. [28] prepared a series of benzimidazole (BIM)-based Cu coordination polymers (CuCPs) as precatalysts by reacting BIM derivatives with Cu_2_O at 60 °C (Figure 2b) to enhance CO_2_RR to C_2+_ products in acidic environments. The decrease or disappearance of the Cu_2_O peaks in X-ray diffraction (XRD) patterns, the disappearance of the Cu-O stretching mode peak in FTIR, and the merging of the N 1S spectra into a single peak in XPS, confirmed the formation of Cu-N bond and the successful preparation of CuCPs. By varying the length of the substituent at the two-position of BIM ligands, a volcano-liked trend in the FE for C_2_+ products was observed. Notably, pentyl-CuCP exhibited the highest FE for C_2_+ products, reaching 73.4 ± 2.9% at a current density of 260 mA cm^−2^.

In addition, organic molecules can be immobilized on metal and carbon substrates using electrografting techniques involving diazotization, allowing organic molecular modifications on the electrode surface. As an instance, after the diazotization of aminopyridine derivatives in HCl solution by using NaNO_2_ to form pyridines-N_2_^+^, in situ reduction in the diazonium cation can be carried out on the surface of the Ag electrode by cyclic voltammetry (CV). The presence of characteristic irreversible reduction peaks in the CV curves indicates that the reduction in diazonium salt generates an aryl radical intermediate, which subsequently bonds covalently to the electrode surface and releases N_2_ gas. As shown in Figure 2c, the cathodic peaks shifted to more negative potentials and the current density decreased during cycles 2 to 5, confirming the successful electrografting of pyridine onto the electrode surface, which was further verified by XPS. The catalyst (Ag-EPy) prepared by this method, with pyridine grafted onto the Ag electrode, shows a significant improvement in product selectivity during the CO_2_ conversion process compared to a single catalyst. A 10-fold activity enhancement is observed at −0.7 V vs. RHE, and demonstrable higher partial current densities for CO are observed, indicating that a cocatalytic effect can be achieved through the integration of the two different catalytic structures [29].

In addition, a facile hydrothermal reaction can also be employed for molecular modification of metal surfaces. Kang et al. [30] applied this approach for constructing a controllable local environment at the molecular scale by grafting amine-functionalized ligand layers onto Ag nanoparticles (NPs) (Figure 2d). In this strategy, 4-aminobutylphosphonic acid (NH_2_BPA) binds to the Ag surface with its phosphonic acid group, while the carbonyl oxygen atoms of the polyvinylpyrrolidone (PVP) molecule also bind to Ag surface atoms preventing the growth of NPs and forming a thin organic layer on the Ag NPs confining NH_2_BPA to the catalyst surface. The results show that the amino groups significantly facilitate CO_2_ adsorption on the nanoparticle surface, while the phosphonic acid groups inhibit the oxygen reduction reaction, and thus the prepared NH_2_BPA-Ag can be used for electroreduction of simulated flue gas with high FE of CO.

### 2.2. Weak Interaction Modification

In addition to the most common metal–organic molecular bonding, many catalysts are modified through weak interactions such as π-π interactions. The π-π interaction modification strategy primarily relies on the interaction between organic molecules with π-electrons, such as aromatic rings and the catalyst. This interaction can modulate the electronic density on the catalyst surface, promote CO_2_ molecule adsorption, or stabilize intermediates, thereby enhancing the reaction selectivity and catalytic efficiency of CO_2_RR.

**Figure 3:**
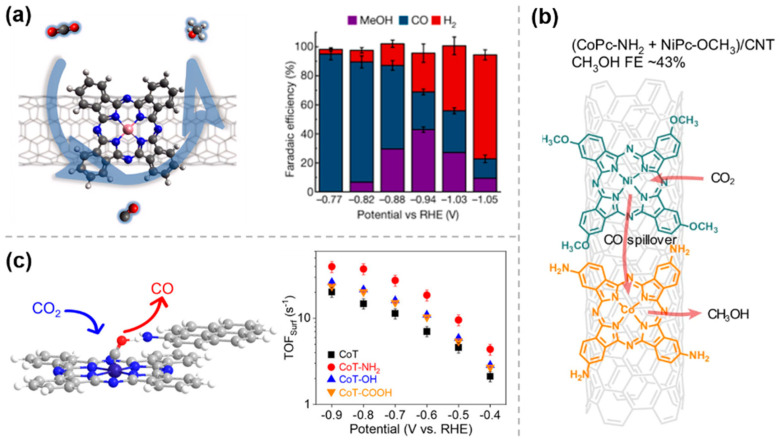
Weak interaction modification by π−π conjugation: (**a**) CoPc/CNTs [31], with permission from Springer Nature, copyright 2019; (**b**) (CoPc−NH_2_ + NiPc−OCH_3_)/CNT [32], with permission from Springer Nature, copyright 2025; and (**c**) CoT−NH_2_ [33], with permission from American Chemical Society, copyright 2025.

Currently, the compounds used for the modification of catalysts through weak interaction are mainly based on phthalocyanine catalysts. Regarding the current problem of poor selectivity in the generation of methanol through CO_2_RR, in 2019, Wang et al. [31] utilized this strategy by uniformly dispersing molecular catalysts such as cobalt phthalocyanine (CoPc) on carbon nanotubes (CNTs) enabled the efficient electrocatalytic reduction of CO_2_ to methanol via a domino process with CO as an intermediate (Figure 3a). Based on this discovery, subsequent studies have explored various modifications of CNTs with CoPc-like materials for CO_2_ electrocatalytic reduction. For example, in order to further improve the efficiency of methanol production from CO_2_RR, the same group [32] reported in early 2025 on a strategy to integrate a second catalytic site into a CoPc-NH_2_/CNT electrocatalyst (Figure 3b). The nickel tetramethoxyphthalocyanine (NiPc-OCH_3_) molecules in this cascade catalyst produce a large amount of CO via CO_2_ reduction, which then overflows to the nearby methanol-active CoPc-NH_2_ sites for further reduction, thereby promoting CO_2_RR to methanol. These studies demonstrate that efficient immobilization of molecular catalysts can be achieved by π-π interactions, which enhance the selectivity of CO_2_RR.

Similarly, 1-aminopyrene molecules can be noncovalently anchored onto a nanosized CoPc nanotube (CoT) via simple π-π interactions, leading to the construction of hangman structures (Figure 3c) [33]. This structure facilitates electron transfer kinetics in both the inner and outer layers of the complex interface and enhances the chemisorption of *COOH and *CO intermediates through the interaction of Co sites and side chain amine groups in a bridging geometry. It therefore improves the intrinsic CO_2_RR activity of CoT and its selectivity for CO.

### 2.3. Polymer-Based Material Modification

Polymers are macromolecular compounds composed of repeating structural units, characterized by their diverse structures, tunable properties, and widespread applications in various fields such as materials science, engineering, and biotechnology. They can be used not only as modifiers for the electrocatalysts to increase surface hydrophobicity and optimize the CO_2_RR process, but also as substrate materials, especially conductive polymers, to receive functionalized modifications from other organic molecules, thereby improving their electrocatalytic performance. The modification of polymer-based material can be primarily achieved through various techniques, including spray-coating, in situ polymerization, and co-electrodeposition.

**Figure 4:**
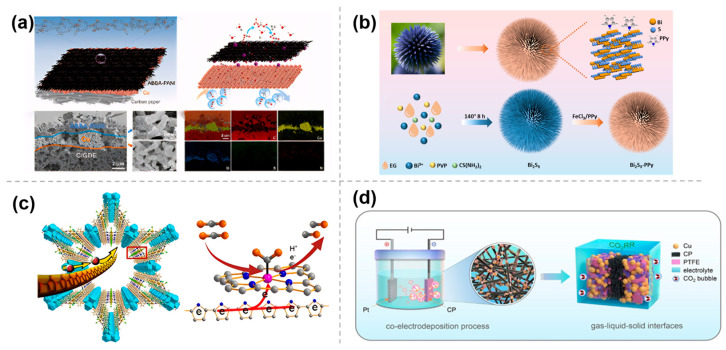
Modification by polymer-based materials: (**a**) Cu/ABSA−PANI [34], with permission from John Wiley and Sons, copyright 2025; (**b**) Bi_2_S_3_−PPy [35], with permission from RSC Publishing, copyright 2025; (**c**) PPy@MOF−545−Co [36], with permission from American Chemical Society, copyright 2021; and (**d**) Cu−PTFE/CP [37], with permission from John Wiley and Sons, copyright 2023.

The spray-coating method allows the easy modification of various pre-prepared polymer molecules onto the substrate material and also facilitates the evaluation of the effect of the polymer layer on the CO_2_RR performance of the catalytic layer. For example, Huang et al. [34] created an ideal microenvironment for CO_2_RR between the Cu catalyst layer and the electrolyte by using polyaniline (PANI) modified by different proton acids (Figure 4a). This polymer layer modulated the local hydrophobicity, facilitated the adsorption and actitation of CO_2_, and also enhanced charge transfer and ion transport at the electrochemical reaction interface, thus significantly improving the acidic CO_2_RR kinetics. In the case of amino benzenesulfonic acid-PANI (ABSA-PANI), the prepared Cu/ABSA-PANI catalysts exhibited both excellent electronic conductivity and ion conductivity, resulting in reduced ohmic losses at the cathode and lower overpotential, achieving FE of 81% for C_2_+ products with a partial current density of 486 mA cm^−2^.

Alternatively, the polymer can also be prepared by in situ polymerization on the electrocatalyst surface. Chen et al. [35] constructed a novel Bi_2_S_3_-polymer polypyrrole (PPy) composite electrocatalyst with rich S vacancies through in situ vapor-phase polymerization with pyrrole on the surface of a 3D hierarchical urchin-like Bi_2_S_3_ nanoflower (Figure 4b). The unique nanostructure morphology maximized the exposure of active sites, thereby increasing the contact area with CO_2_ and improving the adsorption of the reactionintermediate *OCHO, resulting in excellent selectivity for formate. Moreover, the PPy layer on the surface of the Bi_2_S_3_-PPy composite can effectively prevent the loss of S elements and enhance the structural stability of Bi_2_S_3_. In a separate study, Xin et al. [36] inserted the conductive polymer PPy into the channels of MOF-545-Co via in situ polymerization, creating a new composite catalyst, PPy@MOF-545-Co (Figure 4c). In this structure, PPy molecules act like electric cables within the MOF channels, facilitating electron transfer to the Co-TCPP center, which in turn exhibits excellent electrocatalytic performance for CO_2_RR.

In addition, the polymer can be cemented onto the electrode surface by co-electrodeposition to modulate the interfacial microenvironment, especially the construction of hydrophobic microenvironment to overcome the limitation of CO_2_ solubility in aqueous electrolytes. Han et al. [37] reported a direct and facile strategy for polytetrafluoroethylene (PTFE) treatment to construct electrocatalysts by one-step co-electrodeposition of Cu and PTFE on carbon paper. The copper-based electrodes with superhydrophobic surface microenvironment exhibited high ethylene selectivity (Figure 4d). The improved performance was attributed to the formation of a gas–liquid–solid microenvironment, which accelerated mass transfer, increased the local concentration of CO_2_ near the catalyst surface, and improved the adsorption of CO_2_ for the reaction.

## 3. Effects of Molecularly Modified Materials in CO_2_RR

CO_2_RR is a process involving multiple proton-coupled electron transfers, encompassing both electrochemical and chemical reactions. The number of transferred electrons for the main products of CO_2_RR ranges from 2 to 18. This process involves numerous intermediates that significantly influence the reaction pathways and final products. Through molecular modification, the microenvironment of the catalyst surface can be regulated, and new active sites can be created on the catalyst surface, which can enhance the adsorption and activation of CO_2_, lower the reaction barriers, and thereby improve the efficiency of the reduction reaction and the selectivity of the target products. The effects involve multiple factors, including but not limited to the following: the electronic effects of the catalyst, steric hindrance, promoting reactant adsorption and stabilizing intermediates, the hydrophobic environment, and the regulation of pH. The catalytic role of molecularly modified materials in CO_2_RR is mainly reflected in the following aspects.

### 3.1. Electronic Effects

Organic molecules can influence the electronic environment of the catalyst surface, thereby affecting the electron transfer process and altering the catalytic performance. The realization of electronic effects depends on the function of different functional groups in the organic molecules. For example, organic molecules containing electron-donating groups (such as amino, thiol, etc.) can increase the electron density of the catalyst surface by direct electron donation, thereby enhancing the interaction between the catalyst and CO_2_ molecules and promoting effective CO_2_ adsorption. In contrast, certain functional groups with strong electron-withdrawing properties (such as fluoro, carboxyl, etc.) can significantly reduce the electron density on the catalyst surface. The electronic effect not only affects the stability of reaction intermediates but also modifies the reaction pathway to some extent.

**Figure 5:**
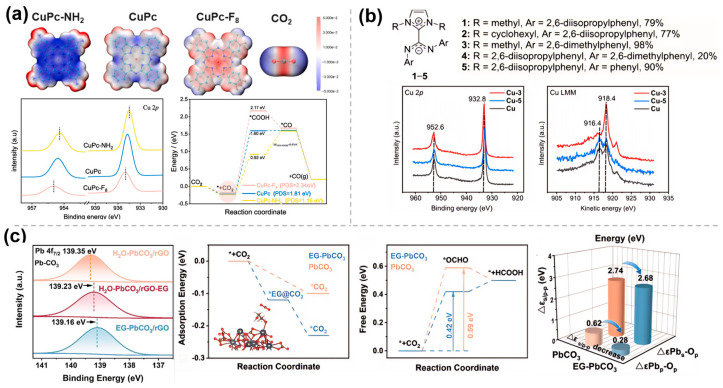
Electronic effects of molecularly modified materials: (**a**) CuPc−NH_2_ [38], with permission from Elsevier, copyright 2025; (**b**) Cu−NHC−CDI [41], with permission from American Chemical Society, copyright 2024; and (**c**) EG−PbCO_3_/rGO [42], with permission from Elsevier, copyright 2025.

As mentioned in Section 2.2, molecular catalysts, such as phthalocyanine catalysts, can effectively catalyze CO_2_RR. Using copper phthalocyanine (CuPc), Peng et al. [38] investigated the effect of the electron-induced action of ligands on the electrocatalytic performance of CO_2_RR by combining electrochemical analysis, in situ characterization, and theoretical calculations (Figure 5a). The results show that the introduction of electron-donating groups (e.g., -NH_2_) increases the contribution of ligand groups to the density near the high electron-occupied states, decreases the width of the HOMO energy gap between CuPc-NH_2_ and CO_2_, and thus decreases the energy barrier formation of *COOH, and thus the FE of CO is close to 100%. On the contrary, the electron-inducing effect of electron-withdrawing groups (e.g., -F) leads to a decrease in the electron density around the Cu center and an increase in the positive electrostatic potential around the Cu-N_4_ site, which facilitates the formation of formic acid by adsorption at the oxygen end of *OCHO. Similarly, the electron donating/withdrawing effects of ligands have been exploited to induce changes in the electronic density at the metal centers of molecular catalysts, such as Cu/CH_2_NH_2_/SWCNTs [39] and MOF (ZIF-7) [40], thereby achieving precise control over catalytic activity, product selectivity, and reaction pathways.

To improve the intrinsic activity of Cu-based materials towards multi-carbon hydrocarbons, Rosas-Hernández et al. [41] exploited the modification of Cu catalysts with N-heterocyclic carbene–carbon diimide (NHC-CDI). XPS measurements indicated that the NHC-CDI ligand 3 (Figure 5b) modified the Cu surface, adjusting its electronic structure, increasing the Cu^0^ concentration, and reducing the Cu^+^ content, thereby enhancing the electron density. Surface charge transfer was assessed using the modified pulse voltammetry method, revealing that the NHC-CDI ligand donates electrons to the Cu surface, stabilizing charged intermediates (e.g., *CO^−^) and enhancing surface charge accumulation. The NHC-CDI modification significantly improved the selectivity and current density for multi-carbon products through electronic effects.

Ren et al. [42] successfully synthesized ethylene glycol-functionalized lead carbonate/ reduced graphene oxide composite (EG-PbCO_3_/rGO) to increase the catalytic conversion rate of Pb-based catalysts. XPS results (Figure 5c) showed that EG modification caused a negative shift in Pb 4f binding energy (Δ = 0.12 eV), with electrons transferred from EG to Pb, thereby reducing its oxidation state. DFT calculations indicated that EG regulated the d-band center of Pb, enhancing orbital hybridization with the *OCHO intermediate and reducing the reaction energy barrier (ΔG decreased by 0.17 eV). Furthermore, EG minimized the difference between the Pb s/p bands and the O p-band, promoting interfacial electron transfer and enhancing catalytic activity.

### 3.2. Steric Hindrance Effects

**Figure 6:**
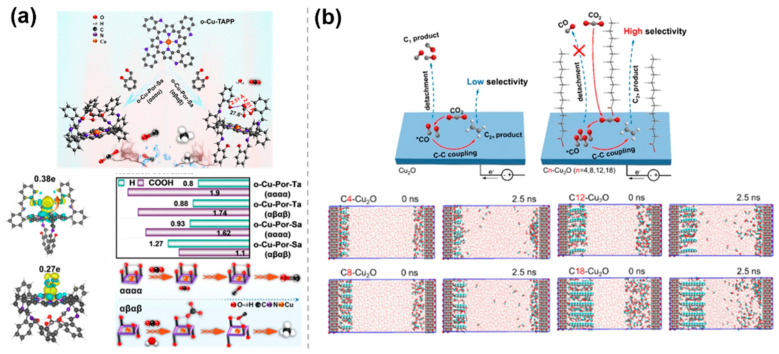
Steric hindrance effects of molecularly modified materials: (**a**) o−Cu−Pro−Sa [43], with permission from John Wiley and Sons, copyright 2025; and (**b**) C_n_−Cu_2_O [44], with permission from American Chemical Society, copyright 2024.

Steric hindrance effect of catalysts, which not only regulates reaction pathways by influencing the adsorption of reactants and the stability of intermediates but also improves catalytic performance by adjusting various factors such as the surface structure of the catalyst, the exposure of active sites, and the stability of the catalyst. Based on the key role of spatial ligand arrangement in inhibiting the competitive HER, Zhang et al. [43] proposed a bifunctional ligand isomerization strategy, which relies on the steric hindrance and hydrogen bonding interactions in o-Cu-Pro-Sa (αααα, αβαβ) to regulate the selectivity of CO_2_RR (Figure 6a). The study revealed the dual role of the steric hindrance effect in the blocked isomer. On the one hand, the symmetric distribution of the -OH group on o-Cu-Por-Sa (αβαβ) generates a steric hindrance effect that inhibits C-C coupling and promotes protonation reactions, leading to the formation of CH_4_. On the other hand, -OH aggregates on o-Cu-Por-Sa (αααα) to form a pocketed hydrogen-bonding lattice, which restricts the free adsorption of CO_2_ and exposes *CO to the formation of CO. To restrict the detachment of C_1_ intermediates and facilitate C-C coupling, Yan et al. [44] proposed a surface-confinement approach by grafting long alkyl chains onto Cu_2_O NPs for CO_2_RR (Figure 6b). The optimized C_12_-Cu_2_O sample exhibited an FE of over 63.0% for C_2_H_4_. In situ electrochemical attenuated total reflection Fourier transform infrared (ATR-FTIR) spectroscopy shows that the signal intensity of the *CO intermediate on the Cn-Cu_2_O samples increased more rapidly during the reaction and decreased more slowly after voltage-off, compared with the original Cu_2_O sample. This suggests that the modified carbon chains limit the diffusion of C_1_ intermediates. Molecular dynamics simulations further confirmed the limiting effect of the alkyl chains as a key factor, resulting in an extended residence time of *CO on C_n_-Cu_2_O, corresponding to a higher chance of C-C coupling, leading to an increase in C_2_+ selectivity.

### 3.3. Promoting Reactant Adsorption and Stabilizing Intermediates

**Figure 7:**
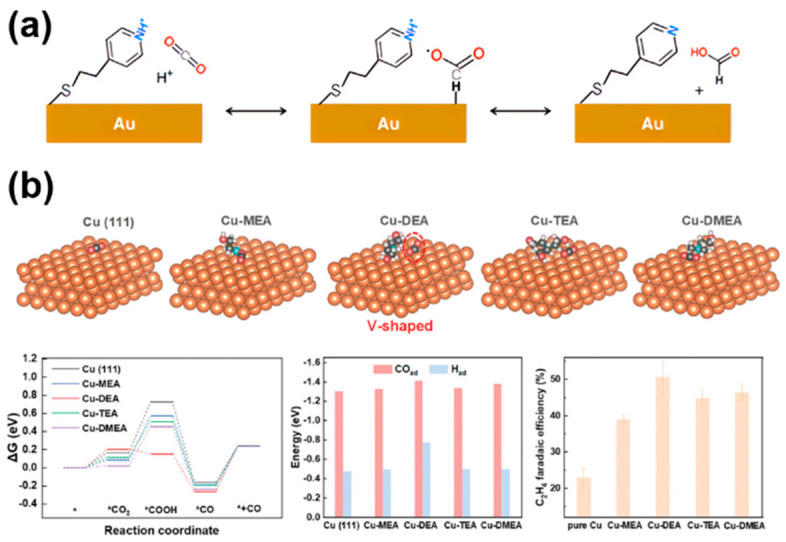
The effect of reactant adsorption and intermediates stabilization in molecularly modified materials: (**a**) Au−PEM [19], with permission from American Chemical Society, copyright 2017; and (**b**) Cu−DEA [45], with permission from John Wiley and Sons, copyright 2024.

In the process of CO_2_RR, the interaction of reactants CO_2_ and intermediates (e.g., *CO, *HCOO, CH_3_O*, etc.) with the catalyst surface is the key to determining the efficiency of the catalytic reaction. Molecularly modified materials can promote the adsorption of CO_2_ or stabilize certain intermediates through molecular functionalization, while maintaining the adsorption of intermediates, which can effectively regulate the selectivity of CO_2_RR and even CORR. Flake et al. [19] carried out CO_2_ electrocatalytic reduction with 4-pyridineethanethiol (4-PEM) on the surface of a functionalized gold (Figure 7a). Compared to gold foil, the FE on the gold electrode modified with 4-PEM was doubled, and the formate yield increased by three times. This was mainly due to the fact that the hydrogen atom in the pyridine ring of 4-PEM interacts with the oxygen atom in the *HCOO intermediate through hydrogen bonding, thereby stabilizing the intermediate and significantly improving formate selectivity. Similarly, the nitrogen atoms in SPy immobilized on the copper surface could stabilize the *HCOO intermediate through hydrogen bonding, greatly enhancing the FE for formate production [18]. Recently, Wang et al. [45] reported a diethanolamine (DEA)-modified Cu (Cu-DEA) catalyst for the reduction of CO_2_ to C_2_H_4_. They utilized theoretical modeling to understand the effects of alkanolamine-modification on the adsorption behavior of CO_2_. DFT calculations showed that the CO_2_ adsorption on Cu-DEA surface transitioned to a V-shaped structure, while CO_2_ maintained a linear structure on the surfaces of other alkanolamine molecules (monoethanolamine (MEA), triethanolamine (TEA), and N, N-dimethylethanolamine (DMEA)) modified Cu (Figure 7b), indicating that the DEA modification promotes CO_2_ activation. Although the energy barrier for CO_2_ adsorption on Cu-DEA is higher than others, the subsequent formation of *COOH intermediates on Cu-DEA possesses self-exothermic reactions, leading to the formation of a large amount of *COOH intermediates. In addition, Cu-DEA has the strongest adsorption energy for *CO and *H, which is beneficial for the C-C coupling and proton coupling step in CO_2_RR process, resulting in good C_2_ selectivity. Briefly, molecular modification of catalysts can modulate the binding ability with CO_2_ molecules or key intermediates, thereby promoting specific reaction pathways to enhance selectivity.

### 3.4. Hydrophobic Environment

**Figure 8:**
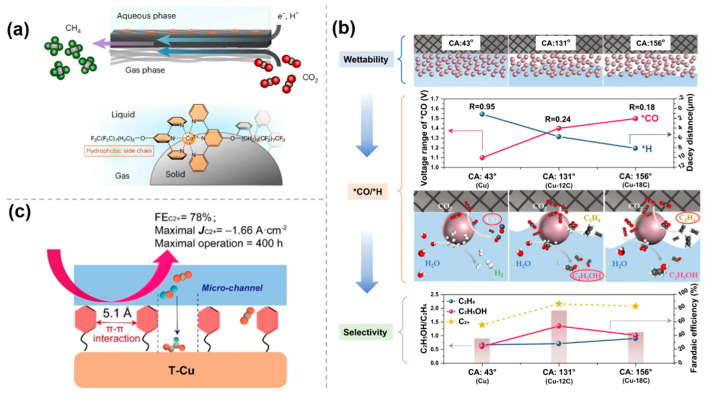
The effect of hydrophobic environment in molecularly modified materials: (**a**) Co Terpy−R_F_ [46], with permission from Springer Nature, copyright 2024; (**b**) Cu−nC [47], with permission from Springer Nature, copyright 2023; and (**c**) toluene−modified Cu nanosheet (T−Cu) [48], with permission from John Wiley and Sons, copyright 2023.

The performance of electrocatalytic CO_2_ reduction reaction is highly dependent on the microenvironment around the cathode, and molecular modifications are crucial to the catalytic process by creating a hydrophobic environment. Upon introduction of hydrophobic molecular modifications, the hydration on the catalyst surface, the adsorption pattern of reactants, and the reaction pathway can be modulated, which significantly improves the selectivity and efficiency of the CO_2_ reduction reaction. Kornienko et al. [46] successfully designed a molecular cobalt terpyridine catalyst with perfluorinated alkyl chains (Co Terpy-R_F_) to assemble the gas–liquid–solid interface. The strong interactions between the perfluoro chains drove the catalyst to assemble in an orderly manner at the gas–liquid–solid interface, forming a hydrophobic microenvironment which can be confirmed by the contact angle (100.7°) of the Co Terpy-R_F_ film. Research has shown that the hydrophobic catalytic pocket can stabilize the intermediates of CO_2_RR, while the pyridine units act as the proton shuttles, transferring protons to active sites and ultimately promoting the production of CH_4_ from CO_2_ (Figure 8a).

To investigate the effect of interfacial wettability on the pathway of CO_2_RR to ethylene and ethanol, Gong et al. [47] proposed to functionalize the Cu surface with alkyl mercaptans with different chain lengths, where the thiol groups coordinated with the substrate to form tunable wettability layers whose hydrophobicity was precisely controlled by the alkyl chain dimensions. The local concentrations of CO_2_ and H_2_O are regulated by different interfacial wettability to achieve the optimal equilibrium of kinetically controlled *CO and *H in a controlled manner. The experimental results show that the interfacial structure changes from a liquid–solid interface to a gas–liquid–solid interface and then to a gas–solid interface with increasing hydrophobicity. Different interfacial structures affect CO_2_ and H_2_O transport, which may lead to changes in *CO and *H coverage. An increase in hydrophobicity will lead to an increase in *CO coverage and a decrease in *H coverage, which will enhance the C-C coupling to form ethylene; conversely, a decrease in hydrophobicity will increase *H coverage and promote the formation of ethanol. Thus, the creation of a gas–liquid–solid interface by forming a suitably hydrophobic surface can enhance ethanol selectivity, whereas a superhydrophobic surface leads to ethylene production (Figure 8b).

To block the transport of interfacial water, Zheng et al. [48] developed a strategy for surface toluene functionalization of Cu nanosheet catalysts. The surface functionalized toluene molecules could self-assemble into ordered hydrophobic microchannels through π-π interactions of hydrophobic and conjugated benzene rings. The most stable distance between these parallel-arrayed benzene rings is 5.1 A, which is larger than that of the long-chain alkyl groups, and can control the transport of water and CO_2_ molecules at the same time. Specifically, the hydrophobic microchannels maintain efficient transport of CO_2_ molecules but delay water supply to the catalyst surface, which in turn facilitates CO_2_ to C_2_+ products (Figure 8c).

### 3.5. Requlation of Interfacial PH

**Figure 9:**
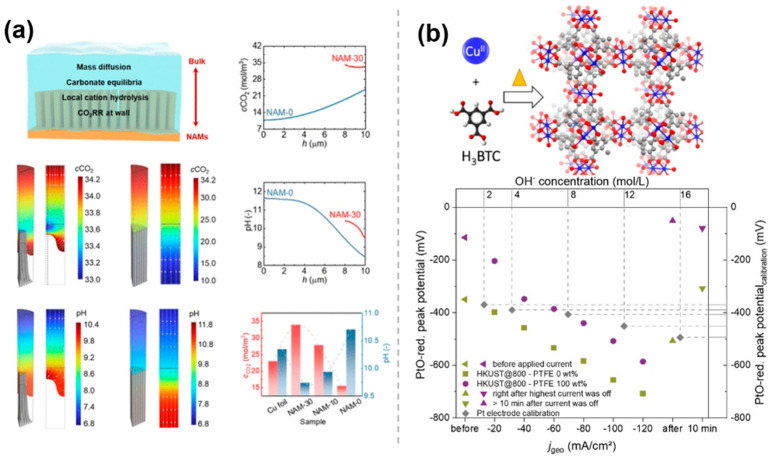
The pH Regulation effect of molecularly modified materials: (**a**) NAMs [49], with permission from American Chemical Society, copyright 2025; and (**b**) Cu_x_O_y_C_z_ [50], with permission from John Wiley and Sons, copyright 2021.

Molecular modifications, in addition to hydrophobicity, play a role in the regulation of the microenvironmental by regulating the interfacial pH. During CO_2_RR, CO_2_ is reduced to various products on the catalyst surface. The formation of these products usually depends on the stability of the intermediates, and the formation and conversion of intermediates are affected by acidity and alkalinity, so the mechanism of the reaction may change under different pH environments. In particular, the CO_2_ electroreduction reaction under acidic conditions can fundamentally solve the problems of CO_2_ loss and salt precipitation and has more favorable reaction kinetics. However, the acidic environment exacerbates the hydrogen precipitation reaction, which is detrimental to the CO_2_RR system. It was shown that modulating the pH around the catalyst by molecular modification constructs could improve the performance of the catalyst in CO_2_RR. Wong et al. [49] designed a tunable superhydrophobic hierarchical Cu nanowire arrays with a microgroove (NAMs), which can be tuned to regulate multiple synergistic effects in the microenvironment by adjusting the structure, including regulating the local pH, stabilizing the microwetting state, increasing the local CO_2_ concentration, and limiting CO intermediates. This process involves the mass transport processes, carbonate species equilibria of OH^–^, CO_2_, HCO_3_^–^, and CO_3_^2–^, the local hydrolysis of K^+^ in the electrolyte phase, and mass consumption and generation during CO_2_RR (top left of Figure 9a). The NAMs exhibit different wetting states, resulting in significant differences in the local CO_2_ concentrations and pH distributions. The computational results of the spatial distributions of the CO_2_ concentration and pH for NAMs with different interfacial structures are shown in Figure 9a. NAM-30 has a stable gas–liquid interface with an electrolyte intrusion depth of about 2 μm, a small local pH gradient (9.6–10.4), and the neutral environment that promotes proton-electron transfer and suppresses HER. However, for NAM-0, the near one-dimensionality and longer diffusion distances lead to a large pH gradient (8.5–11.7), and the alkaline environment exacerbates HER and reduces C_2_^+^ selectivity (Figure 9a). Schuhmann et al. [50] reported a series of Cu_x_O_y_C_z_ nanostructured electrocatalysts derived from Cu-based MOF as a porous self-sacrificial template, providing variations in the structure and composition of the catalysts. The catalysts were blended with polytetrafluoroethylene (PTFE) on gas diffusion electrodes (GDEs) to inhibit the competitive hydrogen precipitation reaction. Since both CO_2_RR and competing HER consume water molecules, this leads to the accumulation of hydroxide ions (OH^−^) locally, thereby altering the local pH value. As the current density increases, the local OH^−^/H_2_O activity ratio significantly increases, indicating an increase in the local pH value (Figure 9b). The addition of PTFE can reduce the electrowetting effect on the electrode surface, thereby inhibiting HER and improving the selectivity of CO_2_RR.

## 4. Summary and Outlook

In this work we have summarized molecular modification strategies for enhancing CO_2_ electroreduction. Specifically, firstly, the strategies for the molecular modification have been summarized, such as metal–organic bonding, weak interactions, and modification with polymer-based materials. Then, the effects of molecularly modified materials on CO_2_RR are enumerated, including electronic effects, steric hindrance effects, the promotion of reactant adsorption and the stabilization of intermediates, the hydrophobic environment and pH regulation.

In the research of molecularly modified materials, possible future directions may include the following: (1) Structure design and optimization. Through advanced techniques such as computational chemistry simulation and machine learning, molecularly modified materials can be designed and optimized more efficiently to improve their catalytic performance in CO_2_RR. This approach can accelerate the discovery of new materials and provide guidance for the structural optimization of catalysts. (2) In-depth study of reaction mechanisms. The in-depth study of the reaction mechanisms of molecular modified materials in CO_2_RR through a combination of experimental and theoretical approaches will help to reveal key steps in the catalytic process and provide a theoretical basis for catalyst design. Particularly, the research to understand electron transfer, reaction pathways, and intermediate stability will be of great importance for catalyst development. (3) The development of multifunctional catalysts by combining different types of molecularly modified materials and developing multifunctional catalysts with synergistic effects may significantly improve the overall performance of CO_2_RR. The study of this new type of catalyst will not only improve catalytic efficiency but also optimize the reaction selectivity and expand the application areas of CO_2_RR.

The application of molecular modification in CO_2_RR enriches the idea of catalyst design and provides an important way to realize efficient and excellent selective CO_2_ conversion. Through different molecular modification strategies, we were able to modulate the surface properties, electronic structures, and reaction mechanisms of the catalysts, thus optimizing the performance of the CO_2_RR. Future work can further explore more efficient and sustainable molecular modification strategies, combined with advanced materials synthesis techniques, to promote the commercial application of CO_2_RR and contribute to solving energy and environmental problems.

## References

[1] Han J., Bai X., Xu X., Husile A., Zhang S., Qi L., Guan J. (2024). Advances and challenges in the electrochemical reduction of carbon dioxide. Chem. Sci..

[2] Shah S.S.A., Javed M.S., Najam T., Molochas C., Khan N.A., Nazir M.A., Xu M., Tsiakaras P., Bao S.J. (2022). Metal oxides for the electrocatalytic reduction of carbon dioxide: Mechanism of active sites, composites, interface and defect engineering strategies. Coordin. Chem. Rev..

[3] Liu X., Liu X.H., Zhang X., Wang H., Zhao Q. (2024). Metal-organic frameworks and their derivatives for electrochemical CO_2_ reduction reaction: Insights from molecular engineering. J. Mater. Chem. A.

[4] Guo W., Liu S., Tan X., Wu R., Yan X., Chen C., Zhu Q., Zheng L., Ma J., Zhang J. (2021). Highly efficient CO_2_ electroreduction to methanol through atomically dispersed Sn coupled with defective CuO catalysts. Angew. Chem. Int. Ed..

[5] Liang Y., Zhao J., Yang Y., Hung S.-F., Li J., Zhang S., Zhao Y., Zhang A., Wang C., Appadoo D. (2023). Stabilizing copper sites in coordination polymers toward efficient electrochemical CC coupling. Nat. Commun..

[6] Zhong D., Fang Q., Du R., Jin Y., Peng C., Cheng D., Li T., Zhao T., Zhang S., Zheng Y. (2025). Selective electrochemical CO_2_ reduction to ethylene or ethanol via tuning* OH adsorption. Angew. Chem. Int. Ed..

[7] Bondue C.J., Graf M., Goyal A., Koper M.T. (2020). Suppression of hydrogen evolution in acidic electrolytes by electrochemical CO_2_ reduction. J. Am. Chem. Soc..

[8] Pan H., Barile C.J. (2020). Electrochemical CO_2_ reduction to methane with remarkably high Faradaic efficiency in the presence of a proton permeable membrane. Energ. Environ. Sci..

[9] Hirunsit P., Soodsawang W., Limtrakul J. (2015). CO_2_ electrochemical reduction to methane and methanol on copper-based alloys: Theoretical insight. J. Phy. Chem. C.

[10] Zhou X., Shan J., Chen L., Xia B.Y., Ling T., Duan J., Jiao Y., Zheng Y., Qiao S.-Z. (2022). Stabilizing Cu^2+^ ions by solid solutions to promote CO_2_ electroreduction to methane. J. Am. Chem. Soc..

[11] Nam D.-H., De Luna P., Rosas-Hernández A., Thevenon A., Li F., Agapie T., Peters J.C., Shekhah O., Eddaoudi M., Sargent E.H. (2020). Molecular enhancement of heterogeneous CO_2_ reduction. Nat. Mater..

[12] Zhang J., Ding J., Liu Y., Su C., Yang H., Huang Y., Liu B. (2023). Molecular tuning for electrochemical CO_2_ reduction. Joule.

[13] Du Z.-Y., Li S.-B., Liang G.-H., Xie Y.-M., Zhang Y., Zhang H., Tian J.-H., Zheng S., Zheng Q.-N., Chen Z. (2024). Promoting water activation via molecular engineering enables efficient asymmetric C–C coupling during CO_2_ electroreduction. J. Am. Chem. Soc..

[14] Wan M., Yang Z., Morgan H., Shi J., Shi F., Liu M., Wong H.-W., Gu Z., Che F. (2023). Enhanced CO_2_ reactive capture and conversion using aminothiolate ligand–metal interface. J. Am. Chem. Soc..

[15] Zhang M.D., Huang J.R., Shi W., Liao P.Q., Chen X.M. (2023). Self-accelerating effect in a covalent-organic framework with Imidazole groups boosts electroreduction of CO_2_ to CO. Angew. Chem..

[16] Yu H., Han X., Hua Z., Yang W., Wu X., Wu Y., Chen S., Hong W., Deng S., Zhang J. (2024). Modulating electronic properties of carbon for selective electrochemical reduction of CO_2_ to methanol on Cu_3_P@C. ACS Catal..

[17] Yang H., Cai H., Li D., Kong Y., Feng S., Jiang X., Hu Q., He C. (2024). Molecular modification enables CO_2_ electroreduction to methane on platinum surface in acidic media. Nat. Sci. Rev..

[18] Creissen C.E., Rivera de la Cruz J.G., Karapinar D., Taverna D., Schreiber M.W., Fontecave M. (2022). Molecular Inhibition for Selective CO_2_ Conversion. Angew. Chem. Int. Ed..

[19] Fang Y., Flake J.C. (2017). Electrochemical reduction of CO_2_ at functionalized Au electrodes. J. Am. Chem. Soc..

[20] Chen J., Liu X., Xi S., Zhang T., Liu Z., Chen J., Shen L., Kawi S., Wang L. (2022). Functionalized Ag with thiol ligand to promote effective CO_2_ electroreduction. ACS Nano.

[21] Souza M.L., Lima F.H.B. (2021). Dibenzyldithiocarbamate-functionalized small gold nanoparticles as selective catalysts for the electrochemical r eduction of CO_2_ to CO. ACS Catal..

[22] Li J.-K., Dong J.-P., Liu S.-S., Hua Y., Zhao X.-L., Li Z., Zhao S.-N., Zang S.-Q., Wang R. (2024). Promoting CO_2_ electroreduction to hydrocarbon products via sulfur-enhanced proton feeding in atomically precise thiolate-protected Cu clusters. Angew. Chem. Int. Ed..

[23] Shen Y., Fang N., Liu X., Ling Y., Su Y., Tan T., Chen F., Lin H., Zhao B., Wang J. (2025). Observation of metal-organic interphase in Cu-based electrochemical CO_2_-to-ethanol conversion. Nat. Commun..

[24] Ding J., Li F., Ren X., Liu Y., Li Y., Shen Z., Wang T., Wang W., Wang Y.-G., Cui Y. (2024). Molecular tuning boosts asymmetric C-C coupling for CO conversion to acetate. Nat. Commun..

[25] Shirzadi E., Jin Q., Zeraati A.S., Dorakhan R., Goncalves T.J., Abed J., Lee B.-H., Rasouli A.S., Wicks J., Zhang J. (2024). Ligand-modified nanoparticle surfaces influence CO electroreduction selectivity. Nat. Commun..

[26] Bhoumik N.C., Padovan Q.A., Akter T., Stem D.K., Barile C.J. (2025). Gold self-assembly on copper electrodes promotes n-propanol formation in electrochemical CO_2_ reduction. Angew. Chem. Int. Ed..

[27] Yue K., Qin Y., Huang H., Lv Z., Cai M., Su Y., Huang F., Yan Y. (2024). Stabilized Cu^0^-Cu^1+^ dual sites in a cyanamide framework for selective CO_2_ electroreduction to ethylene. Nat. Commun..

[28] Deng H., Liu T., Zhao W., Wang J., Zhang Y., Zhang S., Yang Y., Yang C., Teng W., Chen Z. (2024). Substituent tuning of Cu coordination polymers enables carbon-efficient CO_2_ electroreduction to multi-carbon products. Nat. Commun..

[29] Abdinejad M., Irtem E., Farzi A., Sassenburg M., Subramanian S., van Montfort H.-P.I., Ripepi D., Li M., Middelkoop J., Seifitokaldani A. (2022). CO_2_ electrolysis via surface-engineering electrografted pyridines on silver catalysts. ACS Catal..

[30] Liu Z., Yan T., Shi H., Pan H., Kang P. (2024). Grafting amine-functionalized ligand layer on catalyst for electrochemical CO_2_ capture and utilization. Appl. Catal. B Environ..

[31] Wu Y., Jiang Z., Lu X., Liang Y., Wang H. (2019). Domino electroreduction of CO_2_ to methanol on a molecular catalyst. Nature.

[32] Li J., Zhu Q., Chang A., Cheon S., Gao Y., Shang B., Li H., Rooney C.L., Ren L., Jiang Z. (2025). Molecular-scale CO spillover on a dual-site electrocatalyst enhances methanol production from CO_2_ reduction. Nat. Nanotechnol..

[33] Zhou Y., Duan X., Xu X., Win P.E.P., Ren S.-B., Wang J. (2025). Noncovalent construction of hangman cobalt phthalocyanine for enhanced electrochemical carbon dioxide reduction. Chem. Mater..

[34] Su L., Hua Q., Feng G., Yang Y., Mei H., Yu Y., Chang X., Huang Z. (2025). Multifunctional conductive polymer modification for efficient CO_2_ electroreduction in acidic electrolyte. Adv. Funct. Mater..

[35] Li C., Liu Z., Zhou X., Zhang L., Fu Z., Wu Y., Lv X., Zheng G., Chen H. (2023). Bio-inspired engineering of Bi_2_S_3_-PPy composite for the efficient electrocatalytic reduction of carbon dioxide. Energ. Environ. Sci..

[36] Xin Z., Liu J., Wang X., Shen K., Yuan Z., Chen Y., Lan Y.-Q. (2021). Implanting polypyrrole in metal-porphyrin MOFs: Enhanced electrocatalytic performance for CO_2_RR. ACS Appl. Mater. Interf..

[37] Deng T., Jia S., Chen C., Jiao J., Chen X., Xue C., Xia W., Xing X., Zhu Q., Wu H. (2024). Polymer modification strategy to modulate reaction microenvironment for enhanced CO_2_ electroreduction to ethylene. Angew. Chem. Int. Ed..

[38] Fang J., Qin B., Zhang Q., Yang G., Du S., Liu Z., Peng F. (2025). Influence of electron-inducted effect of ligand on electrocatalytic reduction of CO_2_ by copper phthalocyanine. Chem. Eng. J..

[39] Wang K., Huang K., Wang Z., An G., Zhang M., Liu W., Fu S., Guo H., Zhang B., Lian C. (2025). Functional group engineering of single-walled carbon nanotubes for anchoring copper nanoparticles toward selective CO_2_ electroreduction to C_2_ products. Small.

[40] Chen J., Wang G., Dong Y., Ji J., Li L., Xue M., Zhang X., Cheng H.-M. (2024). Controlling the polarity of metal-organic frameworks to promote electrochemical CO_2_ reduction. Angew. Chem. Int. Ed..

[41] Kolding K.N., Bretlau M., Zhao S., Ceccato M., Torbensen K., Daasbjerg K., Rosas-Hernández A. (2024). NHC-CDI ligands boost multicarbon production in electrocatalytic CO_2_ reduction by increasing accumulated charged intermediates and promoting *CO dimerization on Cu. J. Am. Chem. Soc..

[42] Shu Y., Wang Z., Song Z., Wang W., Wang X., Chen Z., Ren Z. (2025). Microenvironment modulation induced by ethylene-glycol modification enables high activity in selective CO_2_ electroreduction over lead-based catalysts. Chem. Eng. J..

[43] Wang H., Ma C., Lu Q., Gu M., Jiang L., Hao Y., Hu F., Li L., Wang G., Peng S. (2025). Precise tuning of functional group spatial distribution on porphyrin rings for enhanced CO_2_ electroreduction selectivity. Angew. Chem. Int. Ed..

[44] Li W., Chen Y., Guo C., Jia S., Zhou Y., Liu Z., Jiang E., Chen X., Zou Y., Huo P. (2024). Confined intermediates boost C_2_^+^ selectivity in CO_2_ electroreduction. ACS Catal..

[45] Lv Z., Wang C., Liu W., Liu R., Liu Y., Feng X., Yang W., Wang B. (2024). Enhanced CO_2_ adsorption and conversion in diethanolamine-Cu interfaces achieving stable neutral ethylene electrosynthesis. Adv. Energy Mater..

[46] McKee M., Kutter M., Wu Y., Williams H., Vaudreuil M.-A., Carta M., Yadav A.K., Singh H., Masson J.-F., Lentz D. (2025). Hydrophobic assembly of molecular catalysts at the gas-liquid-solid interface drives highly selective CO_2_ electromethanation. Nat. Chem..

[47] Lin Y., Wang T., Zhang L., Zhang G., Li L., Chang Q., Pang Z., Gao H., Huang K., Zhang P. (2023). Tunable CO_2_ electroreduction to ethanol and ethylene with controllable interfacial wettability. Nat. Commun..

[48] Liu Z., Lv X., Kong S., Liu M., Liu K., Zhang J., Wu B., Zhang Q., Tang Y., Qian L. (2023). Interfacial water tuning by intermolecular spacing for stable CO_2_ electroreduction to C_2_^+^ products. Angew. Chem. Int. Ed..

[49] Cheng Y., Li Q., Salaman M.I.B., Wei C., Wang Q., Ma X., Liu B., Wong A.B. (2025). Microenvironment tailoring for electrocatalytic CO_2_ reduction: Effects of interfacial structure on controlling activity and selectivity. J. Am. Chem. Soc..

[50] Sikdar N., Junqueira J.R.C., Dieckhöfer S., Quast T., Braun M., Song Y., Aiyappa H.B., Seisel S., Weidner J., Öhl D. (2021). A metal-organic framework derived Cu_x_O_y_C_z_ catalyst for electrochemical CO_2_ reduction and impact of local pH change. Angew. Chem. Int. Ed..

